# Isoenergetic reduction of dietary macronutrients affects body composition, physical activity, and post-prandial hormone responses in lean and obese cats fed to maintain body weight

**DOI:** 10.3389/fvets.2025.1588330

**Published:** 2025-05-19

**Authors:** Hannah Godfrey, Érico de Mello Ribeiro, Shoshana Verton-Shaw, Anna Kate Shoveller, Darcia Kostiuk, Janelle Kelly, Jennifer Saunders Blades, Adronie Verbrugghe

**Affiliations:** ^1^Department of Biomedical Sciences, University of Guelph, Guelph, ON, Canada; ^2^Department of Clinical Studies, University of Guelph, Guelph, ON, Canada; ^3^Department of Animal Biosciences, University of Guelph, Guelph, ON, Canada; ^4^Champion Petfoods Holding Inc., Morinville, AB, Canada

**Keywords:** dietary protein, dietary carbohydrate, dietary fat, feline nutrition, dual energy X-ray absorbsiometry, actigraphy, leptin, ghrelin

## Abstract

**Introduction:**

High consumption of dietary carbohydrates (nitrogen-free extract, NFE) in extruded dry foods is postulated as a risk factor for feline obesity, though evidence is limited. This study utilized a three-test diet approach to isolate the effect of each macronutrient on body composition, voluntary physical activity, and serum satiety hormone response in lean and obese cats.

**Materials and methods:**

A pairwise isoenergetic reduction as % metabolizable energy (ME, using modified Atwater) of dietary macronutrients created a low-protein (LP; protein = 28%, fat = 40%, NFE = 32%ME), low-fat (LF; protein = 40%, fat = 30%, NFE = 30%ME), and low-carbohydrate (LC; protein = 36%, fat = 41%, NFE = 23%ME) diet. Cats (lean *n* = 9; obese *n* = 9) were fed the LP, LF, or LC diet to maintain body weight for 4-weeks in a 3 × 3 Latin square design. Voluntary physical activity was measured from day 15–21. On day 23, body composition was assessed by dual energy x-ray absorptiometry. On day 24, blood was collected for fasted and 6-h post-prandial serum satiety hormones. Data were analysed using SAS Studio via proc. GLIMMIX with significance set at *p* < 0.05.

**Results:**

Greater lean soft tissue mass was observed for LF compared to LP and LC (*p* = 0.0101) though no other changes in body composition were observed. Daily food intake (g/d) differed among diets (LP: 56.14 ± 0.56; LF: 59.52 ± 0.59; LC: 56.50 ± 0.56; *p* = 0.0003) though energy intake (kcal/d) was similar (LP: 218.47 ± 2.32; LF: 220.42 ± 2.34; LC: 225.37 ± 2.39; *p* = 0.1076). Voluntary physical activity was similar for lean and obese cats (*p* = 0.4025). LP cats exhibited less physical activity during dark hours compared to LF and LC (*p* = 0.0155). No body condition effect was observed for serum leptin or ghrelin (*p* = 0.6243 and *p* = 0.6747). Obese cats on LP exhibited a lower serum leptin iAUC compared to obese cats on LC (P_BC*Diet_ = 0.0093). Post-prandial serum ghrelin decreased in all cats whereas serum peptide YY (PYY) increased at 1-, 2-, and 3-h post-prandial (P_Time_ = 0.0094 and P_Time_ = 0.0050). Serum ghrelin was lower at 6-h post-prandial (P_Diet*Time_ = 0.0030) and serum PYY was greater at 1-h post-prandial (P_Diet*Time_ = 0.0086) for LC.

**Discussion:**

Overall, the LP, LF, and LC diets were not associated with differences in most parameters, supporting that cats are metabolically flexible if nutrient requirements are met. There may be benefits regarding satiety hormone responses to a LC diet in cats though more research is required.

## 1 Introduction

Extruded dry foods for cats are often high in dietary carbohydrates (referring to nitrogen-free extract, NFE), containing up to 40% on a metabolizable energy (ME) basis when using Modified Atwater factors (1, 2). In contrast, a typical prey species for a feral or wild cat, such as a mouse, contains approximately 2%ME NFE (3). Since cats are obligate carnivores, having evolved on a diet low in dietary carbohydrates, the consumption of extruded dry foods, and by extension, greater carbohydrates, has been postulated as a risk factor for feline obesity (1, 4); although, there is no consensus among scientists and clinicians. Obesity in cats is the most common nutritional disorder, posing a major health and welfare concern (5, 6). However, high fat diets (protein, 33% ME; fat 64% ME; NFE, 3% ME and protein, 32% ME; fat 40% ME; NFE, 28% ME, respectively) appear to drive greater food and energy intake, body weight (BW), and body fat mass (BFM) compared to high carbohydrate diets (protein, 33% ME; fat 9% ME; NFE, 57% ME and protein, 31% ME; fat 27% ME; NFE, 42% ME, respectively) when fed ad libitum (7, 8). Further, cats have continuously demonstrated a “carbohydrate ceiling” of 300 kJ/d (9). At this level, cats appear to limit their daily energy intake (8–10). The “carbohydrate ceiling” may explain the greater energy intake in cats when offered high protein or high fat compared to high carbohydrate foods (10–12). Indeed, a recent meta-analysis could not detect a correlation between increasing dietary carbohydrate intake and body fat mass (BFM) in cats (13). However, the primary outcomes for these studies are focused on food and energy intake as well as body composition when fed ad libitum (7, 8). Gooding et al. (14) evaluated a high fat (protein, 34% metabolizable energy [ME]; fat, 30% ME, NFE, 25.8% ME) and a high carbohydrate diet (protein, 30% ME; fat, 11% ME; NFE, 47.1% ME) and found that both diets reduced voluntary physical activity.

Verbrugghe et al. (15) incorporated a three-test diet approach wherein a pairwise reduction of dietary macronutrients was used to isolate the individual effect of each macronutrient on the glucose and insulin response in cats. Using this approach, we previously found that cats consuming a low-carbohydrate (LC; protein 35%ME, fat 40%ME, NFE 20%ME) diet exhibited a lower fasted respiratory quotient (RQ) compared to cats consuming a low-fat (LF; protein 40%ME, fat 27%ME, NFE 27%ME) diet and a low-protein (LP, protein 28%ME, fat 40%ME, NFE 28%ME) diet induced a lower heat increment of feeding compared to the LF diet when measured over a 20 h period (16). No differences in energy expenditure were observed in this study (16). A lower fasted RQ could indicate greater fatty acid oxidation which may be favourable in reducing BFM and improving skeletal muscle mitochondrial capacity and reducing the heat increment of feeding (17, 18). This proof-of-principle study aims to utilize the same three-test diet approach to further build upon these findings. As such, this study will test the effect of a LP, LF, and LC diet on voluntary physical activity, body composition, and the serum satiety hormones leptin, ghrelin, glucagon-like peptide-1 (GLP-1), gastric inhibitory polypeptide (GIP), and peptide YY (PYY) in lean and obese cats fed to maintain BW for 4 weeks. This objective aims to test the following hypotheses, first, when fed to maintain BW, macronutrient distribution, above requirements, does not affect body composition in cats; however, body composition is different between lean and obese cats as obese cats have higher total tissue mass (TTM), BFM, and greater lean soft tissue mass (LSTM) to compensate for the excess weight compared to lean cats. Second, a reduction in dietary fat results in greater physical activity due to a reduction of cholecystokinin release in both lean and obese cats (19, 20), though obese cats are less physically active than lean cats (21, 22). Third, post-prandial satiety response is less pronounced with obesity, though fasted leptin and ghrelin are positively associated with obesity (23–25). Last, a reduction in dietary protein subsequently results in a lower post-prandial satiety hormone response in cats.

## 2 Materials and methods

All experimental procedures were approved by the University of Guelph Animal Care Committee (AUP #4865) and were in accordance with national and institutional guidelines for the care and use of animals in research.

### 2.1 Animals

Eighteen purpose-bred (Marshall’s Bio Resources, Waverly, NY, USA), domestic shorthair male, neutered colony cats were enrolled. Cats were between 3 and 4 years of age at the time of enrollment. Using a 9-point body condition score (BCS) scale (26), cats with a BCS of 4 (lean) or 5 (ideal) out of 9 were grouped as “lean” (*n* = 9, BCS = 4 or 5 out of 9) and cats with a BCS of 8 or 9 (obese) out of 9 were grouped as “obese” (*n* = 9, BCS = 8 or 9 out of 9) by two assessors with 100% similarity of scores (H.G., S.V.S.). The mean (±SEM) BW for the lean and obese groups were 4.38 (±0.09) kg and 5.80 (±0.08) kg, respectively. Besides the incidence of obesity in nine cats, all animals enrolled in the study were deemed healthy based on a veterinary physical examination, complete blood count, and serum biochemical profile.

### 2.2 Housing

Cats were grouped housed in a free-living environment (7.0 m x 5.8 m) at the Animal Biosciences Cat Colony at the University of Guelph (Guelph, ON, Canada). The cats had a 12-h light and dark cycle with lights turning on at 0700 h and off at 1900 h. Temperature was maintained at 24.3 ± 0.05°C and humidity maintained at 40.3 ± 0.9% throughout the trial. The room was cleaned and sanitized daily, and litter boxes scooped twice per day. Cats had access to scratching posts, cat trees of differing size and height, hiding boxes, beds, perches, and various toys for enrichment. In addition, cats were provided with human interaction in the form of voluntary brushing, petting, and playing for a maximum of 2 h per day.

### 2.3 Dietary treatments and feeding

Prior to the study, during a period of 4 weeks, cats were fed a commercially available, extruded dry diet for adult maintenance (Orijen Original Cat, Champion Petfoods, Edmonton, AB, Canada). For the study, three extruded dry diets were formulated specifically for this research study for adult maintenance in accordance with the Association of American Feed Control Officials (AAFCO) (27) (Champion Petfoods LP, Morinville, AB, Canada). The test diets were formulated with the same ingredients in varying quantities in an isoenergetic approach (15) to create a pairwise change in macronutrient content (% ME) forming a LP, LF, and LC diet as previously described (16) and summarized in Table 1. The LC diet differed from the LF and the LP diets by isoenergetic substitution of carbohydrate for fat and carbohydrates for protein, respectively, using the modified Atwater factors as per guidelines set by the Association of American Feed Control Officials (AAFCO) (27, 28). All three test diets were formulated to have similar vitamin and mineral concentrations, total dietary fibre, as well as physical structure.

**Table 1:** Ingredient composition and proximate analysis (% as fed) of a low-protein (LP), low-fat (LF), and low-carbohydrate (LC) test diets formulated for adult maintenance using an isoenergetic approach with varying levels of the same ingredients fed to cats (*n* = 18) for 4 weeks in a 3×3 Latin square design when offered to maintain body weight.

Parameter	LP	LF	LC
Ingredients (%)			
Chicken meal	17.6	18	18.4
Fresh chicken	20.0	5.0	8.0
Dried chicken	1.1	17.1	14.5
Pea starch	19.8	17.8	6.8
Pea fibre	10.9	12.0	11.9
Egg powder	6.0	2.0	8.0
Poultry hydrolysate	0.5	7.7	8.0
Oat groats	7.5	10.6	9.7
Chicken fat	6.8	2.2	6.0
Fish oil	2.1	0.7	1.9
Dry palatant	1.3	1.3	1.3
Liquid palatant	3.0	3.0	3.0
Vitamin/mineral premix	2.7	1.7	1.7
Kelp	0.3	0.5	0.4
Salt	0.4	0.4	0.4
Proximate analysis (% as fed)			
Moisture	8.25	5.22	6.05
Dry Matter	91.75	94.78	93.95
Crude Protein	31.24	42.48	40.94
Crude Fat	18.4	12.9	19.10
Crude Fibre	4.40	4.80	4.70
Ash	6.30	7.40	7.20
Total Dietary Fibre	11.20	11.80	12.40
NFE^1^	31.41	27.20	22.01
Metabolizable Energy^2^ (kcal/kg)	3,756.75	3,535.30	3,826.75
Macronutrient distribution (% ME)			
Protein	28.0	40.0	36.0
Fat	40.0	30.0	41.0
NFE	32.0	30.0	23.0

LC, low-carbohydrate; LF, low-fat; LP, low-protein; ME, metabolizable energy; NFE, nitrogen-free extract.

1 Calculated as: Nitrogen-Free Extract = 100 − (Crude Protein+Crude Fat + Crude Fiber + Ash) (31).

2 Calculated as: ME = 10*(Crude Protein*3.5) + (Crude Fat*8.5) + (NFE*3.5) (31).

A proximate analysis was performed on each of the three diets (Bureau Veritas, Mississauga, ON, Canada), summarized in Table 1, using the appropriate methods outlined by the Association of Official Analytical Chemists (AOAC) and the American Oil Chemist Society (AOCS) (29, 30). Included in these measures were moisture and dry matter (AOAC 935.29), crude protein via the Kjeldahl method (AOAC 992.15), crude fat using gas chromatography (AOAC 922.06, AOAC 933.05), and crude fibre and ash via combustion (AOCS Ba 6a-0.5 and AOAC 923.03, respectively). Additionally, total dietary fiber was analysed (AOAC 991.43, 985.29). The following equations were used to calculated NFE and ME (31):


$$NFE\;\left(\%\right)=100-\left(\begin{array}{l} \mathrm{Crude}\;\mathrm{Protein}\;\left(\%\right)-\mathrm{Crude}\;\mathrm{Fat}\;\left(\%\right)-\\ \mathrm{Crude}\;\mathrm{Fibre}\;\left(\%\right)-\mathrm{Ash}\left(\%\right)-\mathrm{Moisture}\left(\%\right) \end{array}\right)$$

$$\mathrm{ME}=10*\left[\left(\mathrm{Crude}\;\mathrm{Protein}*3.5\right)+\left(\mathrm{Crude}\;\mathrm{Fat}*8.5\right)+\left(NFE*3.5\right)\right]$$

All diets were offered to the cats once daily (0800 h) for 1 h in individual enclosed crates (82 cm x 55 cm x 55 cm) to measure individual food and energy intake throughout the study. Food was offered at an amount to meet each cats’ individual maintenance energy requirement determined using historical colony data prior to the start of the study (where BW was consistent for 4-weeks) and adjusted as necessary through weekly assessment of BW.

### 2.4 Experimental design

Cats were assigned to one of three groups, with equal distribution of lean (*n* = 9) and obese (*n* = 9) cats and thus, balanced for BW and BCS. Following a 3×3 Latin square design, each group received each test diet for 4 weeks (28 days) in a random order. Test diets were offered at an amount to meet each cats’ individual maintenance energy requirement determined using historical colony data and adjusted as necessary. Daily food intake (DFI) was recorded throughout the study and daily energy intake (DEI) (kcal/d) was calculated by multiplying the ME (kcal/g) by the DFI (g/day). Weekly BW was measured (Defender™ 3,000 Xtreme W scale, OHAUS®, Parsippany, NJ, USA) and BCS were assessed (H.G., S.V.S). Sampling and data collection procedures were performed from day 14 to day 24 of each test period. On day 15, cats were fitted with a harness and an ActiGraph™ monitor to measure voluntary physical activity for 6 days (day 15–day 21). On day 23 of each test period, jugular catheters were placed under anesthesia. Immediately following, cats were maintained under sedation to assess body composition by dual-energy x-ray absorptiometry (DXA). Blood collection occurred on day 24 for one pre-prandial (0750 h) and six post-prandial blood samples (1, 2, 3, 4, 5, and 6 h postprandial) for determination of serum satiety hormones leptin, ghrelin, GLP-1, GIP, and PYY.

### 2.5 Physical activity

Voluntary physical activity was measured over 7 days (day 15–day 21) using ActiGraph™ activity monitors (ActiGraph™ LLC, Penascola, FL, USA) that were fitted to a harness for which cats had previously been acclimated to. To reduce variability, cats wore the same activity monitor at each period. The ActiLife® software (ActiLife® v9.0.0, ActiGraph™ LLC, Penascola, FL, USA) was used to convert data into arbitrary values referred to as activity counts defined over 10 s time periods (epochs). Converted data was then used to calculate total weekly physical activity, total weekly light and total weekly dark physical activity counts, as well as the mean daily physical activity and mean daily light and dark physical activity counts, and the light:dark ratio. Light periods corresponded to the period from 0700 h to 1900 h, whereas dark periods corresponded to the period from 1900 h to 0700 h.

### 2.6 Catheter placement

On day 23, cats were sedated via intramuscular injection of butorphanol (0.3 mg/kg BW at 10 mg/mL) (Zoetic, Kirkland, QC, Canada) and dexmedetomidine (0.005 mg/kg at 0.5 mg/mL) (Dexdomitor, Zoetis, Kirkland, QC, Canada). Once sedated, an intravenous catheter was placed in the cephalic vein for intravenous propofol (Fresenius Kabi Canada Ltd., Richmond Hill, ON, Canada) (1–4 mg/kg) induction. To facilitate central jugular catheter placement, propofol was administered intravenously with a maximum total dose of 8 mg/kg BW (10 mg/mL). For catheter placement, the area over one jugular vein was aseptically prepared. The jugular catheter (MILA International Inc., Florence, KY, USA) was inserted and secured. Catheter size varied dependent on cat. Heparinized saline solution (10 IU/mL) was used to prime both proximal and distal catheter lines to maintain patency. Reversal via atipamezole (Zoetic, Kirkland, QC, Canada) (5 mg/mL) was administered intramuscularly (0.02 mg/kg) after cats completed individual DXA scans.

### 2.7 Body composition

On day 23 of each test period, following jugular catheter placement, sedation was maintained and a fan-beam DXA device (Prodigy® Advance GE Healthcare, Madison, WI, USA) was used to determine body composition. Cats were positioned in ventral dorsal position with forelimbs extended cranially. Three scans were taken over approximately 15-min periods using Small Animal Mode and the average was used to estimate TTM, BFM, whole-body fat % (BF%), LSTM, bone mineral content (BMC) and bone mineral density (BMD), obtained from the system software (enCORE Version 16; GE Healthcare, Madison, WI, USA). When three scans could not be taken, the average was determined using two scans. Following DXA, sedation was reversed using 0.2 mg/kg BW of atipamezole (Antisedan, Zoetis, Kirkland, QC, Canada) (5 mg/ml).

### 2.8 Blood collection and analyses

On day 24 of each period, after an 18-h fast, one fasted blood sample (0750 h) and six hourly post-prandial blood samples were collected after consumption of the respective test diet (0800 h). To isolate serum, whole blood was collected via jugular catheter (BD Vacutainer® Venous Blood Collection Tubes: Serum tubes, Becton Dickson, Franklin Lakes, NJ, USA). Fasted and post-prandial samples (1.5 ml) of whole blood were collected in individual blood tubes containing the DPP-IV inhibitor (Millipore Sigma, Billerica, MA, USA), protease inhibitor (Sigma-Aldrich, St. Louis, MO, USA), and Pefabloc SC inhibitor (Sigma-Aldrich, St. Louis, MO, USA) to prevent enzymatic degradation of satiety hormones before centrifugation and isolation of serum for leptin, ghrelin, GLP-1, GIP, PYY. Samples were placed on ice and allowed to clot prior to centrifugation at 2,500 rpm for 15 min at 4°C (LegendRT, Kendro Laboratory Products 2002, Germany). Serum was separated via pipette and aliquoted into Fisherbrand™ Microcentrifuge Tubes (Thermo Fisher Scientific, Rochester, NY, USA) and stored at −20°C until further analyses.

Commercially available ELISA kits were used to analyze serum satiety hormones in duplicate. Leptin was analyzed using a feline-specific ELISA kits (Limit of Detection, LOD, 0.1 ng/ml; Cat Leptin ELISA Kit Product # MBS057075; MyBioSource LLC, San Diego, CA, USA). The Ghrelin (Rat, Mouse) EIA Kit (LOD, 0.12 ng/ml; Product # EK-031-31; Phoenix Pharmaceuticals Inc., Phoenix, AZ, USA) which has previously been validated for use in cats (32) was used to assess serum ghrelin. Three ELISA kits validated for use in cats were used to measure GLP-1, GIP, and PYY: GLP-1 amide EIA Kit (LOD, 0.2 ng/mL; Product # S-1359.0001; Peninsula Laboratories, Inc., San Carlos, CA, USA), Peptide YY EIA Kit (LOD, 0.65 ng/ml; Product # S-1274.0001; Peninsula Laboratories, Inc., San Carlos, CA, USA), and Cat GIP ELISA Kit (LOD, 5.0 pg./ml; Product # MBs064115-96; MyBioSource LLC, San Diego, CA, USA), respectively. Each kit was run according to the protocols provided by the manufacturer and evaluation of quality control samples. Intra-assay reliability of ELISA kits was 7.2, 6.40, 9.9, 8.9, and 8.8% for ghrelin, leptin, GLP-1, PYY, and GIP, respectively. Absorbances were read and standard curves were generated using BioTek® Absorbance Reader with Gen5™ software and standard curves were generated using BioTek Gen5 software (Agilent Technologies, Inc., Santa Clara, CA, USA). The coefficients of variation (CV), or the ratio of the standard deviation to the mean, were assessed for each set of duplicates. A CV of <20% was deemed acceptable and the average was used for further analysis.

### 2.9 Statistical analysis

The statistical analysis of all data was carried out using SAS Studio 3.8 (SAS Institute, Cary, NC, USA). Data are presented as least squared mean ± SEM with significance set as *p* < 0.05. Area under the curve (AUC) data for serum satiety hormones, were calculated using SAS Studio via the trapezoidal method. Normality of the residuals was assessed via the Shapiro–Wilk test and log transformation was used as needed to meet the assumptions of ANOVA. Data were analyzed using the proc. GLIMMIX procedure as a repeated measures of variance (ANOVA) model, with body condition and test diet, as well as their interaction, as the fixed effect, period as the random effect and cat as the subject. A Tukey post-hoc adjustment using the covariance structure (autoregressive, compound symmetry, variance components, or unstructured) that resulted in the smallest Akaike information criterion value was used to separate means when the fixed effects were significant.

## 3 Results

The three test diets were consumed and tolerated by all cats with no adverse effects. One cat from the lean group was removed due to behavioural issues reducing the lean group sample size to eight cats. Cats were assigned to lean or obese groups via BCS; however, due to a large discrepancy between the assigned BCS and the DXA BF% results, one cat from the obese group was removed from statistical analyses, reducing the obese group to a sample size of eight cats.

### 3.1 Daily intakes

Obese cats were observed to have a greater DFI (g/d), DEI (kcal/d), and greater intake of all three macronutrients (g/d) (*p* < 0.0001); however, on per kg BW basis, there was no difference in DFI (g) or DEI (kcal) between lean and obese cats (*p* = 0.3875) (Table 2). Regardless of body condition, cats consuming the LP and LC diet had a lower DFI compared to cats consuming the LF diet on a per day and a per kg BW basis (*p* = 0.0003 and *p* < 0.0001, respectively). However, DEI (kcal/d) was similar across all three diets (*p* = 0.108). Regardless of body condition, DEI (kcal/kg BW) was greater for cats consuming the LC food compared to LP or LF (*p* < 0.0001); however, no differences were observed for the interaction between body condition and diet (*p* = 0.1439). Protein intake was highest for cats consuming the LF diet and lowest for cats consuming the LP (*p* < 0.0001). Cats consuming the LF had the lowest fat intake compared to cats consuming the LP or LC diet (*p* < 0.0001) and cats consuming the LC diet had the lowest NFE intake, followed by the LP diet and the LF diet (*p* < 0.0001). No significant interaction of body condition and diet were observed for DFI, DEI, or intake of protein, fat, or NFE (Supplementary Table 1).

**Table 2:** Mean daily energy intake (DEI) of lean (*n* = 8) and overweight (*n* = 8) cats and of cats consuming a low-protein (LP, *n* = 16), low-fat (LF, *n* = 16), or low-carbohydrate (LC, *n* = 16) test diet for 4 weeks in a Latin square design.

Parameter	Body condition		Test diet			*p*-values	
	Lean (*n* = 8)	Obese (*n* = 8)	LP (*n* = 16)	LF (*n* = 16)	LC (*n* = 16)	P_BC_	P_Diet_
DFI (g/day)	49.72 ± 0.42^B^	66.19 ± 0.56^A^	56.14 ± 0.56^b^	59.52 ± 0.59^a^	56.50 ± 0.56^b^	<0.0001	0.0003
DFI (g/kg BW)	11.84 ± 0.38	11.33 ± 0.39	11.38 ± 0.27^b^	11.94 ± 0.28^a^	11.43 ± 0.27^b^	0.3683	<0.0001
DEI (kcal/day)	192.19 ± 1.76^B^	255.05 ± 2.34^A^	218.47 ± 2.32	220.42 ± 2.34	225.37 ± 2.39	<0.0001	0.1076
DEI (kcal/kg BW)	45.95 ± 1.49	44.02 ± 1.58	44.65 ± 1.09^b^	44.44 ± 1.09^b^	45.88 ± 1.09^a^	0.3875	<0.0001
Protein Intake (g/d)	18.84 ± 0.64^B^	25.08 ± 0.86^A^	17.53 ± 0.43^b^	25.30 ± 0.62^a^	23.13 ± 0.56^a^	<0.0001	<0.0001
Fat Intake (g/d)	31.82 ± 1.15^B^	42.23 ± 1.52^A^	40.18 ± 1.06^a^	28.46 ± 0.73^b^	43.06 ± 1.11^a^	<0.0001	<0.0001
NFE Intake (g/d)	5.01 ± 0.17^B^	6.67 ± 0.23^A^	5.51 ± 0.13^b^	6.88 ± 0.17^a^	5.09 ± 0.12^c^	<0.0001	<0.0001

A,B denotes significant difference for BC along the row; a,b,c denotes significant differences for Diet along the row. No significant interaction of body condition and diet were observed. Values expressed as LSM ± SEM. BC, body condition; DFI, daily food intake; DEI, daily energy intake; LC, low-carbohydrate; LF, low-fat; LP, low-protein; LSM, least square means; SEM, standard error of the mean.

### 3.2 Physical activity

The average daily, light, and dark vector magnitude counts per 10 s epoch are presented in Table 3. No significant differences were observed between lean and obese cats for the average daily physical activity counts (P_BC_ = 0.8873). Additionally, average physical activity counts during light or dark periods did not differ between lean and obese cats (P_BC_ = 0.7591 and P_BC_ = 0.4025, respectively). No diet effect was observed for average daily and light activity counts (P_Diet_ = 0.3148 and P_Diet_ = 0.4516, respectively); however, the LC and LF diets resulted in greater dark physical activity compared to the LP diet (P_Diet_ = 0.0155). There were no interaction effects between body condition and diet for daily, light, and dark physical activity counts (P_BC*Diet_ = 0.6365, P_BC*Diet_ = 0.4526, and P_BC*Diet_ = 0.6568, respectively). The light:dark physical activity ratio was greater for lean cats compared to obese cats (P_BC_ = 0.0436) and a trend was observed for the light:dark physical activity ratio wherein it tended to be greater for cats consuming the LP diet than for cats consuming the LF and LC diets (P_Diet_ = 0.0570). No interaction of body condition and diet occurred (P_BC*Diet_ = 0.9631).

**Table 3:** Mean voluntary daily (24-h), light (0700–1900 h), and dark (1900–0700 h) vector magnitude counts per 10 s epoch of lean (*n* = 8) and overweight (*n* = 8) cats and of cats consuming a low-protein (LP, *n* = 16), low-fat (LF, *n* = 16), or low-carbohydrate (LC, *n* = 16) test diet for 4 weeks in a Latin square design.

Counts per 10 s epoch	Body condition		Test diet			*p*-values	
	Lean (*n* = 8)	Obese (*n* = 8)	LP (*n* = 16)	LF (*n* = 16)	LC (*n* = 16)	P_BC_	P_Diet_
Daily Physical Activity	56.70 ± 7.45	58.23 ± 7.45	56.55 ± 5.41	56.56 ± 5.41	59.29 ± 5.41	0.8873	0.3418
Light Physical Activity	77.75 ± 9.68	73.47 ± 9.68	76.85 ± 7.14	72.99 ± 7.14	76.99 ± 7.14	0.7591	0.4516
Dark Physical Activity	35.75 ± 5.66	42.67 ± 5.66	36.28 ± 4.12^b^	40.18 ± 4.12^a^	41.17 ± 4.12^a^	0.4025	0.0155
Light:Dark Activity	2.30 ± 0.15^A^	1.79 ± 0.15^B^	2.20 ± 0.12	2.02 ± 0.12	1.91 ± 0.12	0.0436	0.0570

A,B denotes significant differences for BC along a row and a,b denotes significant differences for Diet along a row. No significant interaction of body condition and diet were observed. Values expressed as LSM ± SEM. BC, body condition; LC, low-carbohydrate; LF, low-fat; LP, low-protein; LSM, least square means; SEM, standard error of the mean.

### 3.3 Body composition

Weekly BW remained consistent throughout the study. Mean (±SEM) BW was 4.27 ± 0.07 kg for lean cats and 5.82 ± 0.07 kg for obese cats (P_BC_ < 0.0001). Weekly BW did not differ among diets (LP = 5.05 ± 0.05 kg; LF = 5.05 ± 0.05 kg; LC = 5.04 ± 0.05 kg; P_Diet_ = 0.8929). The TTM, BFM, BF%, LSTM, BMC, and BMD results from DXA for lean and obese cats are summarized in Table 4. Obese cats were observed to have a greater TTM (P_BC_ < 0.0001), BFM (P_BC_ < 0.0001), BF% (P_BC_ < 0.0001), and LSTM (P_BC_ < 0.0001). Additionally, obese cats in the present study had greater BMC compared to lean cats (P_BC_ = 0.004), though BMD was not different (P_BC_ = 0.310). No difference was observed between test diets for TTM (P_Diet_ = 0.1276), BFM (P_Diet_ = 0.4711), or BF% (P_Diet_ = 0.2712). Cats consuming the LF diet had greater LSTM compared to cats consuming LP or LC (P_Diet_ = 0.0101). Both BMC and BMD were similar between the LP, LF, and LC groups (P_Diet_ = 0.2016 and P_Diet_ = 0.2662, respectively). Interaction effects of body condition and test diet did not occur (Supplementary Table 1).

**Table 4:** Dual-energy x-ray absorptiometry measurements of lean (*n* = 8) and obese (*n* = 8) cats and of cats consuming a low-protein (LP, *n* = 16), low-fat (LF, *n* = 16), or a low-carbohydrate (LC, *n* = 16) test diet for 4 weeks in a Latin square design.

	Body condition		Test diet			*p*-value	
	Lean (*n* = 8)	Obese (*n* = 8)	LP (*n* = 16)	LF (*n* = 16)	LC (*n* = 16)	P_BC_	P_Diet_
TTM (g)	4,096.57 ± 66.77^B^	5,668.67 ± 66.77^A^	4,874.82 ± 48.80	4,906.92 ± 48.75	4,866.11 ± 48.80	<0.0001	0.1276
BFM (g)	617.33 ± 67.10^B^	1,486.17 ± 67.10^A^	1,063.72 ± 48.51	1,044.14 ± 48.47	1,047.38 ± 48.51	<0.0001	0.4711
BF (%)	15.02 ± 1.26^B^	26.20 ± 1.26^A^	20.55 ± 0.92	20.36 ± 0.92	20.93 ± 0.92	<0.0001	0.2712
LSTM (g)	3,487.04 ± 80.76^B^	4,174.76 ± 80.76^A^	3,815.92 ± 58.71^b^	3,875.44 ± 58.71^a^	3,801.35 ± 58.71^b^	<0.0001	0.0101
BMC (g)	181.86 ± 6.42^B^	213.29 ± 6.42^A^	197.81 ± 4.56	196.81 ± 4.56	198.11 ± 4.56	0.004	0.2016
BMD (g/cm^2^)	0.48 ± 0.01	0.50 ± 0.01	0.49 ± 0.01	0.49 ± 0.01	0.49 ± 0.01	0.310	0.2662

A,B denotes significant difference for BC along the row; a,b,c denotes significant differences for Diet along the row. No significant interaction of body condition and diet were observed. Values expressed as LSM ± SEM. BC, body condition; BFM, body fat mass; BF%, body fat percent; BMC, bone mineral content; BMD, bone mineral density; LC, low-carbohydrate; LF, low-fat; LP, low-protein; LSM, least square means; LSTM, lean soft tissue mass; SEM, standard error of the mean; TTM, total tissue mass.

### 3.4 Satiety hormones

**Figure 1:**
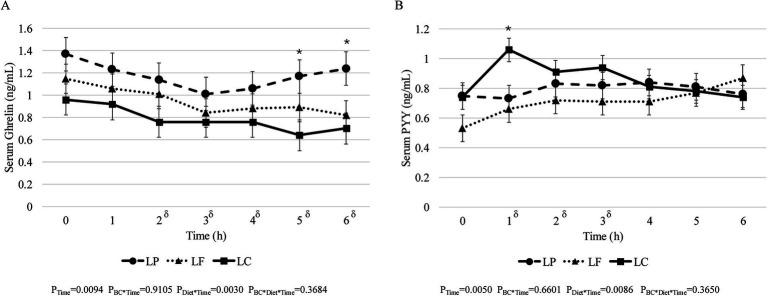
Six hours post-prandial serum concentrations of **(A)** ghrelin and **(B)** PYY in adult cats consuming a low-protein (LP, *n* = 16; dashed line •), low-fat (LF, *n* = 16; dotted line ▲), or low-carbohydrate (LC, *n* = 16; solid line ■) diet for 4 weeks in a Latin square design. *δ* denotes significant Time effect. *Denotes significant diet effect (*p* < 0.05). Values expressed as LSM ± SEM.

There were no significant effects of body condition, diet, or their interaction on fasted leptin (P_BC_ = 0.6747, P_Diet_ = 0.8014, P_BC*Diet_ = 0.2568), ghrelin (P_BC_ = 0.6243, P_Diet_ = 0.2051, P_BC*Diet_ = 0.2742), GLP-1 (P_BC_ = 0.6448, P_Diet_ = 0.6749, P_BC*Diet_ = 0.4601), GIP (P_BC_ = 0.7774, P_Diet_ = 0.7628, P_BC*Diet_ = 0.0686), and PYY (P_BC_ = 0.8545, P_Diet_ = 0.1025, P_BC*Diet_ = 0.3005) concentrations (Supplementary Table 2). Similarly, the iAUC was not different between lean and obese cats, diet, or their interaction for ghrelin (P_BC_ = 0.6446, P_Diet_ = 0.3492, P_BC*Diet_ = 0.3276), GLP-1 (P_BC_ = 0.7712, P_Diet_ = 0.4462, P_BC*Diet_ = 0.3622), GIP (P_BC_ = 0.7762, P_Diet_ = 0.7395, P_BC*Diet_ = 0.2006), and PYY (P_BC_ = 0.6577, P_Diet_ = 0.4995, P_BC*Diet_ = 0.0686) (Supplementary Table 2). According to the post-hoc adjustment the iAUC for serum leptin concentrations was greater for lean cats consuming the LP diet (1.32 ± 0.66 ng/ml × h) and obese cats consuming the LC diet compared (1.39 ± 0.57 ng/ml × h) to obese cats consuming the LP diet (−1.43 ± 0.57 ng/ml × h) (P_BC*Diet_ = 0.0093); though no other differences were observed (P_BC_ = 0.3438, P_Diet_ = 0.2734, P_BC*Diet_ = 0.0093). Regardless of body condition or diet, there was no effect of time on serum GLP-1 (P_Time_ = 0.1193), leptin (P_Time_ = 0.5179), or GIP (P_Time_ = 0.9842). Post-prandial ghrelin concentrations decreased over time from fasted in all cats regardless of body condition or diet (P_Time_ = 0.0094) and cats consuming the LC diet had lower serum ghrelin concentrations at 5- and 6-h post-prandial compared to both the LP and LF diet (P_Diet*Time_ = 0.0030) (Figure 1A); however, no effect of body condition or the interaction of body condition and diet was observed on post-prandial ghrelin concentrations (P_BC*Time_ = 0.9105 and P_BC*Diet*Time_ = 0.3684, respectively). Serum PYY concentrations were greater at 1-, 2-, and 3-h post-prandial compared to fasted concentrations (P_Time_ = 0.0050) regardless of body condition and diet (Figure 1B). Cats consuming the LC diet exhibited greater serum PYY concentrations compared to cats consuming the LF and LP diet at 1-h post-prandial (P_Diet*Time_ = 0.0086); though no effect of body condition or the interaction between body condition and diet was observed (P_BC*Time_ = 0.6601 and P_BC*Diet*Time_ = 0.3650, respectively). In addition, no effect of body condition, diet, or their interaction was observed on post-prandial serum leptin (P_Bc*Time_ = 0.9157, P_Diet*Time_ = 0.9647, P_BC*Diet*Time_ = 0.2775), GLP-1 (P_BC*Time_ = 0.4575, P_Diet*Time_ = 0.8135, P_BC*Diet*Time_ = 0.8881), or GIP (P_BC*Time_ = 0.8773, P_Diet*Time_ = 0.9773, P_BC*Diet*Time_ = 0.0888) concentrations (data not shown).

## 4 Discussion

Research has shown that when fed ad libitum, high fat diets appear to be a bigger risk factor for greater energy intake in cats, and, thus, an increase in BW driven by BFM (7, 8). However, in the present study, cats were fed to maintain BW, and TTM was not affected by diet. Gooding et al. (14) previously observed that long-term feeding (85 days) of a high fat diet when fed to energy requirements resulted in an increase in BW and BFM compared to a high carbohydrate diet. Additionally, a trend towards a decline in LSTM and BMC was observed in cats in that study consuming the high fat diet. In the present study, which was of shorter duration (28 days), diets did not affect TTM, BFM, BMC, or BMD; however, cats consuming the LF diet exhibited greater LSTM when fed to maintain BW. Gooding et al. (14) hypothesized that the changes in body composition were due to changes in fat and carbohydrate intakes. In the present study, cats consuming the LF diet had the greatest dietary protein and NFE intakes (g/d). High carbohydrate diets in endurance trained dogs can increase resting muscle glycogen stores (33). A preliminary study in cats showed that consumption of a high carbohydrate diet compared to consumption of a high protein diet, resulted in greater accumulation of hepatic and muscle glycogen stores in the fed state (34). Hoenig et al. (35) observed an increase in glycogen synthesis and an RQ greater than 1 in cats following insulin infusion via euglycemic hyperinsulinemic clamp. We previously reported a greater fasted, immediate post-prandial, and post-absorptive respiratory quotient (RQ) in cats consuming a LF diet of similar ingredients and macronutrient composition to the present study (protein 40%ME, fat 28%ME, NFE 32%ME) after 2 weeks (16). Although both insulin response and glycogen content were not measured in the present study, an increase in glycogen stores would be captured by DXA within the LSTM which could be the reason for this finding. However, research investigating dietary macronutrient content on glycogen synthesis and deposits in LSTM is limited and requires further investigation. Additionally, a greater RQ is associated with greater reliance on carbohydrate oxidation, or mixed macronutrients compared to a low RQ which is indicative of fatty acid oxidation (17). The greater RQ with a LF diet (16) could suggest that the shift to greater carbohydrate oxidation preserves fat stores and adjusts gluconeogenesis from proteins thereby conserving amino acids for protein synthesis and protein turnover for muscle mass, rather than energy production. The LF diet had the greatest NFE and protein—and therefore amino acid—intake. Taken together, less reliance on amino acids for gluconeogenesis and greater amino acid intake allows for amino acids to be more readily available for protein synthesis, potentially contributing to greater LSTM.

Interestingly, differences in total voluntary physical activity were not observed; however, there were changes in light and dark activity. Cats were fed once a day at 0800 h in the present study, therefore, lower dark physical activity could be due to a reduction of food seeking behaviours in cats consuming the LP diet. Consistent with previous work in laboratory cats, physical activity was greater during light than dark hours (36). However, anticipatory behaviour of colony cats has previously been documented prior to lights-on and food renewal (37), similar to Beagle dogs (38). Additionally, serum ghrelin concentrations were greater in cats consuming the LP diet at 5- and 6-h post-prandial. Ghrelin is an orexigenic hormone that promotes hunger and is suppressed after consumption of a meal (39). Greater ghrelin concentrations may suggest increased hunger; however, voluntary food intake and satiation was not measured directly in the present study, and it is important to note that the satiety hormone response is limited to the 6-h post-prandial response, whereas 24-h responses could be of interest for future research to further explore satiety and food seeking behaviour to further elucidate the changes in light and dark physical activity.

The lower dark physical activity counts observed in cats consuming the LP diet could be attributed to the carbohydrate and fat content of this diet. Both a high fat (protein, 34% metabolizable energy [ME]; fat, 30% ME, NFE, 25.8% ME) and a high carbohydrate (protein, 30% ME; fat, 11% ME; NFE, 47.1% ME) diets were previously observed to reduce voluntary physical activity in cats (14). In humans, dietary carbohydrate intake appears to increase tryptophan and, thus, serotonin levels (40). Tryptophan competes with large neutral amino acids (LNAA) for access to the blood–brain barrier transporters for the synthesis of serotonin (41, 42). The LP diet cumulates into a greater tryptophan:LNAA ratio due to the greater carbohydrate content and a reduction in crude protein, and therefore, a reduction in LNAA. This, in turn, induces feelings of lethargy and tiredness (42, 43). Additionally, this can lead to a decrease in adrenaline release resulting in changes in mood and reduced energy (42, 44). A reduction in physical activity, alertness, and increased feeling of tiredness in response to dietary fats has also been observed in humans (19, 45). The reduced activity in response to dietary fats could be due to greater cholecystokinin secretion following high fat consumption (19, 20) which can result in sleepiness, reduced alertness, and reduced appetite (46, 47). Additionally, high dietary fat intake may influence tryptophan availability for serotonin. Indeed, free fatty acids will bind to albumin in circulation reducing the albumin available to bind to tryptophan (48, 49). Albumin-bound tryptophan has reduced capacity to access the blood–brain barrier transporters; therefore, a greater free fatty acid concentration in response to high dietary fat intake could further increase tryptophan and serotonin resulting in reduced physical activity (48, 49). From human studies, the type of carbohydrate or fat may also be important. Alternatively, omega-3 fatty acids and other fatty acid types have been shown to improve mood and increase physical activity (50, 51). Further, vitamins and minerals such as thiamine, iron, and vitamin D, C, and E, can also play a role in mood, and potentially, physical activity (52). Diets in the present study were formulated to have similar vitamin and mineral content; however, in order to achieve the pairwise reduction of macronutrients, this required changes in some ingredient quantities. This can result in slight differences between vitamin and mineral concentrations, although all diets were formulated above AAFCO recommended nutrient levels. Due to the lower energy content of the LF diet, cats consumed a greater volume of food, and this could also increase the intake of these nutrients compared to the LP and LC diets. Future studies should investigate specific fatty acid and other nutrient types to elucidate the role of diet on physical activity in cats. Further, although a reduction of physical activity during dark hours in response to a LP diet was observed in the present study, it was not sufficient to affect total physical activity. Therefore, minimal to no benefits with regards to obesity prevention and treatment via changes to physical activity may be achieved with a LP diet. However, reducing physical activity during dark hours, without reducing total physical activity, could be beneficial to pet owners who view nighttime activity as a behavioural problem (53).

As mentioned previously, a reduction in ghrelin concentrations are expected following a meal (39) which was supported in the present study where, regardless of diet, all cats exhibited a post-prandial reduction in ghrelin concentrations. Alternatively, leptin is an anorexigenic hormone that signals the hypothalamus, as well as peripheral tissues such as adipocytes and skeletal muscle, to inhibit food intake and regulate BW (39). Previous studies have not found an effect of macronutrient intake on post-prandial leptin concentrations (54, 55) and no difference in serum leptin concentrations was observed between lean and obese cats in the present study, suggesting that these cats were not yet leptin resistant (8). However, obese cats consuming the LP diet exhibited an overall reduction in serum leptin concentrations according to the iAUC, whereas obese cats consuming a LC diet and lean cats consuming the LP diet both had an overall increase in serum leptin. It is unclear what these findings mean; however, a relationship between insulin and leptin exists wherein insulin stimulates leptin synthesis and secretion (56). The present study did not assess insulin or insulin sensitivity; though amino acids are more potent stimulators of insulin secretion than glucose in cats (57, 58). Consuming a LP diet will subsequently reduce amino acid intakes compared to the LF or LC diets. A reduction of circulating insulin could limit leptin production and secretion (56). Additionally, the low iAUC was observed specifically for obese cats consuming the LP which could indicate alterations in the sensitivity to glucose stimulated insulin secretion in obese cats. These findings warrant further investigation of the interaction between body condition and diet on satiety hormones in cats.

In humans, dietary fat is the primary stimulator for PYY, GLP-1, and GIP (59, 60). A potential cause for the lack of differences in post-prandial GLP-1 and GIP response to dietary macronutrient compositions in the present study could be that the change in macronutrient density with each diet was not sufficient to elicit detectable differences. A previous study was unable to detect differences in cats when consuming a test diet with 20% greater protein content and 42% reduced fat content compared to a control diet (61) whereas differences in GIP and GLP-1 secretion in cats have been observed when individual bolus or oral loads of amino acids, lipids, or glucose have been administered (62, 63). Similar to McCool et al. (61), these findings suggest that although individual dietary macronutrients may alter secretion of GIP or GLP-1, these effects may be opposing and therefore, the overall sum of the effect is unable to elicit a response. Further, this study observed large variability between cats for serum satiety hormones, though all serum satiety hormones in the present study were within ranges previously observed in cats (23, 64–67). Large variability between cats has previously been observed and was attributed to a small sample size (*n* = 12) (54). This variability between cats may have been, in part, a potential factor for the lack of differences observed; however, this is likely multifactorial.

Regarding PYY, an increase in the first 2-h post-prandial following consumption of a meal is consistent with post-prandial curves in cats from Camara et al. (65). A dearth of data exists in cats regarding post-prandial PYY; however, the present study suggests that diets higher in protein or fat elicit a greater response on immediate PYY secretion than carbohydrates. Overall, the LC diet in the present study appeared to maintain a lowered ghrelin secretion at 5- and 6-h post-prandial and elicited a greater peak in PYY secretion post-prandially. Due to the role of PYY on energy homeostasis, such as reducing voluntary food intake (68–70) and the role of ghrelin in hunger (39), the findings from the present study suggests that compared to the LP and LF diet, the LC diet may be beneficial in satiety hormone response and requires further investigation.

A surprising finding from the present study was the lack of difference in physical activity between lean and obese cats suggesting that a reduction of physical activity occurs in response to obesity rather than a predisposing risk factor in these colony cats. Important to note that activity counts were in line with previously published values (21, 65, 71) though slight variations are expected due to changes in physical activity with age, sex, neuter status, and monitor location. Differences between lean and obese or overweight cats have previously been observed (21, 22). However, BF% was not measured in the previous studies, and discrepancies in BCS and BF% have previously been observed (72–74) and so the BF% of cats in previous studies may be greater than the BF% observed in the present study. Similar to a previous study using colony cats, BCS as assessed by trained individuals corresponded to 26.2% for obese cats (BCS 8 or 9 out of 9) and 15.0% for lean cats (BCS 4 or 5 out of 9) in the present study (72). Further, TTM, BFM, BF%, and LSTM were all greater in the obese cats compared to lean cats in the present study. However, cats in the present study have fluctuated in BCS due to the various nature of studies they were previously enrolled in (67, 75–77). As such, the obese cats in the present study had been of obese body condition for approximately 6-months prior to enrollment in the study. Therefore, duration of excess BFM may have affected the findings. However, the difference in the light to dark ratio between lean and obese cats in the present study suggests that there are differences in the pattern of physical activity. One hypothesis could include that the lean cats sustained longer bouts of physical activity, for example, during social enrichment hours, whereas obese cats may have had reduced capacity for longer bouts; therefore, more shorter bouts of activity occurred. Further, the shift could be due to disruptions in sleep patterns and sleep disturbances with obesity which has been observed in humans (78, 79). The effect of obesity on sleep patterns and quality in cats has not been investigated to the authors’ knowledge. Additionally, cats were on a 12-h light/dark cycle and feeding occurred at 0800-h each morning, and so a slight shift in activity during the dark hours for obese cats could be indicative of food anticipatory behaviors. However, no differences in fasted ghrelin, the hunger hormone, was observed between lean and obese cats. The lack of differences observed for fasted and post-prandial ghrelin between lean and obese cats could be due to the lack of differences in serum leptin concentrations. One of the many roles of leptin includes the suppression of ghrelin; therefore, obese individuals who are leptin resistant will have an inability of leptin to suppress ghrelin secretion (80), likely resulting in the greater concentration of ghrelin previously observed in obese cats (24). However, if cats in the present study are not yet leptin resistant, this could explain our findings. Indeed, cats in the present study were young (2–3 years of age) and, as discussed, have had fluctuations in body composition. Additionally, although the BF% of the obese cats in the present study was significantly different from the lean cats, the BF% was lower than that of cats in previous studies. Backus et al. did not observe a change in circulating leptin concentrations when the BW of cats increased by 10% and concluded that the cats had not yet developed leptin resistance (8). To our knowledge, the duration of an obese condition and the associated changes that can occur in circulating leptin and ghrelin in cats are not well understood.

As a proof-of-principle study that aimed to test the use of a pairwise, isoenergetic reduction of dietary macronutrients to produce a LP, LF, and LC diet on various parameters in cats, the present study used a short-term feeding design. Longer term effects (~13 weeks) of high levels of dietary macronutrients have resulted in differences in responses compared to short term (~2–4 weeks) effects in cats (14). Although this study concludes that this methodology is safe and effective in cats over a short-term period, this method should be used in a long-term study to further elucidate how macronutrients may affect body composition, satiety hormones, and physical activity in cats. The present study sheds new light on the role of dietary macronutrient distributions in feline health and metabolism. These findings do not support a negative role of dietary carbohydrate intakes over a short duration of 4-weeks in feline health when fed to energy balance. Rather, there may be a potential benefit of carbohydrate inclusion of 30% ME and protein at 40% ME as shown by the greater LSTM with the LF diet. This may be particularly important with regards to obesity treatment through weight loss since this process can result in loss of both BFM and LSTM (81, 82); however, a study incorporating a similar design during weight loss is needed. The duration and magnitude of obesity may also be an important factor in satiety hormone responses and differences and should be further explored.

## Data Availability

The original contributions presented in the study are included in the article/Supplementary material, further inquiries can be directed to the corresponding author.
