# Supplementary material for: Isoenergetic reduction of dietary macronutrients affects body composition, physical activity, and post-prandial hormone responses in lean and obese cats fed to maintain body weight
Source: Front Vet Sci. 2025 May 19;12:1588330. doi: 10.3389/fvets.2025.1588330 (PMC12128646; doi:10.3389/fvets.2025.1588330)
Supplement: Supplementary file 1 [file Table_1.docx]

**Supplementary Table 1.** Mean daily food, energy, and macronutrient intakes, dual energy x-ray absorptiometry measurements of lean cats consuming the low protein (LP, *n=8*) low fat (LF, *n*=8), or a low carbohydrate (LC, *n*=8) test diet and obese cats consuming the LP (n=8), LF (n=8), or LC (n=8) test diet for 4 weeks in a Latin square design

|  |  | **LC** | **LF** | **LP** | **P_BC*Diet_** |
| --- | --- | --- | --- | --- | --- |
| **Intakes** | | | | | |
| **DFI (g/d)** | *Lean* | 48.78 ±0.69 | 51.93 ±0.73 | 48.51 ±0.68 | 0.7086 |
|  | *Obese* | 65.43 ±0.92 | 68.23 ±0.96 | 64.96 ±0.91 |  |
| **DEI (kcal/d)** | *Lean* | 194.65 ±2.93 | 192.24 ±2.89 | 189.71 ±2.85 | 0.7977 |
|  | *Obese* | 260.95 ±3.92 | 252.72 ±3.79 | 251.57 ±3.79 |  |
| **Protein Intake (g/d)** | *Lean* | 19.97 ±0.69 | 22.10 ±0.76 | 15.15 ±0.52 | 0.1057 |
|  | *Obese* | 26.79 ±0.92 | 28.98 ±1.0 | 20.29 ±0.70 |  |
| **Fat Intake (g/d)** | *Lean* | 37.18 ±0.56 | 24.80 ±0.37 | 34.91 ±0.52 | 0.7977 |
|  | *Obese* | 49.85 ±0.75 | 32.60 ±0.49 | 46.30 ±0.70 |  |
| **NFE Intake (g/d)** | *Lean* | 4.40 ±0.06 | 6.00 ±0.08 | 4.76 ±0.07 | 0.7086 |
|  | *Obese* | 5.90 ±0.08 | 7.88 ±0.11 | 6.37 ±0.10 |  |
| **Dual Energy X-Ray Absorptiometry** | | | | | |
| **TTM (g)** | *Lean* | 4078.58 ±68.88 | 4113.33 ±68.86 | 4097.79 ±68.86 | 0.7210 |
|  | *Obese* | 5653.65 ±68.86 | 5700.52 ±68.86 | 5651.84 ±68.88 |  |
| **BFM (g)** | *Lean* | 624.26 ±68.50 | 618.08 ±68.50 | 609.65 ±68.50 | 0.6837 |
|  | *Obese* | 1503.18 ±68.50 | 1470.21 ±68.50 | 1485.11 ±68.50 |  |
| **BF (%)** | *Lean* | 15.30 ±1.30 | 14.93 ±1.30 | 14.84 ±1.30 | 0.6983 |
|  | *Obese* | 26.56 ±1.30 | 25.78 ±1.30 | 26.26 ±1.30 |  |
| **LSTM (g)** | *Lean* | 3456.83 ±83.04 | 3517.41 ±83.04 | 3489 ±83.04 | 0.4830 |
|  | *Obese* | 4145.86 ±83.04 | 4233.47 ±83.04 | 4144.96 ±83.04 |  |
| **BMC (g)** | *Lean* | 182.07 ±6.45 | 180.96 ±6.45 | 192.53 ±6.45 | 0.5899 |
|  | *Obese* | 214.14 ±6.45 | 212.65 ±6.45 | 213.08 ±6.45 |  |
| **BMD (g/cm2)** | *Lean* | 0.48 ±0.01 | 0.48 ±0.01 | 0.48 ±0.01 | 0.8980 |
|  | *Obese* | 0.50 ±0.01 | 0.49 ±0.01 | 0.50 ±0.01 |  |

No significant interaction of body condition and diet were observed.

Values expressed as LSM±SEM

BC, body condition; BFM, body fat mass; BF%, body fat percent; BMC, bone mineral content; BMD, bone mineral density; DFI, daily food intake; DEI, daily energy intake; LC, low carbohydrate; LF, low fat; LP, low protein; LSM, least square means; LSTM, lean soft tissue mass; SEM, standard error of the mean; TTM, total tissue mass
