# Supplementary material for: Isoenergetic reduction of dietary macronutrients affects body composition, physical activity, and post-prandial hormone responses in lean and obese cats fed to maintain body weight
Source: Front Vet Sci. 2025 May 19;12:1588330. doi: 10.3389/fvets.2025.1588330 (PMC12128646; doi:10.3389/fvets.2025.1588330)
Supplement: Supplementary file 2 [file Table_2.docx]

**Supplementary Table 2.** Fasted satiety hormone concentrations and iAUC of lean (*n*=8) and obese (*n*=8) cats and of cats consuming a low protein (LP, *n*=16), low fat (LF, *n*=16), or a low carbohydrate (LC, *n*=16) test diet for 4 weeks in a Latin square design

|  | **Body Condition** | | **Test Diet** | | | **P-Values** | | |
| --- | --- | --- | --- | --- | --- | --- | --- | --- |
|  | **Lean (*n*=8)** | **Obese (*n*=8)** | **LP (*n*=16)** | **LF (*n*=16)** | **LC** **(*n*=16)** | **P_BC_** | **P_Diet_** | **P_BC*Diet_** |
| **Fasted Concentrations** | | | | | | | | |
| **Leptin (ng/mL)** | 2.66 ±2.46 | 1.69 ±0.85 | 2.04 ±1.40 | 2.20 ±1.70 | 2.18 ±2.36 | 0.6747 | 0.8014 | 0.2568 |
| **Ghrelin (ng/mL)** | 1.26 ±0.23 | 1.14 ±0.23 | 1.45 ±0.26 | 1.18 ±0.23 | 0.97 ±0.24 | 0.6243 | 0.2051 | 0.2742 |
| **GLP-1 (ng/mL)** | 0.90 ±0.60 | 0.82 v0.62 | 0.78 ±0.48 | 0.85 ±0.73 | 0.96 ±0.64 | 0.6448 | 0.6749 | 0.4601 |
| **GIP (ng/mL)** | 38.08 ±22.50 | 35.99 ±18.83 | 38.15 ±19.95 | 35.89 ±14.61 | 37.41 ±26.50 | 0.7774 | 0.7628 | 0.0686 |
| **PYY (ng/mL)** | 0.63 ±0.10 | 0.65 ±0.10 | 0.73 ±0.08 | 0.58 ±0.09 | 0.61 ±0.08 | 0.8545 | 0.1025 | 0.3005 |
| **iAUC** | | | | | | | | |
| **Ghrelin (ng/mL x h)** | -0.58 ±0.39 | -0.88 ±0.49 | 0.19 ±0.68 | -1.10 ±0.68 | -1.28 ±0.68 | 0.6446 | 0.3492 | 0.3276 |
| **GLP-1 (ng/mL x h)** | -0.27 ±0.89 | 0.14 ±1.02 | 0.43 ±0.79 | -0.65 ±0.88 | 0.02 ±0.83 | 0.7712 | 0.4462 | 0.3622 |
| **GIP (ng/mL x h)** | 5.81 ±14.71 | -0.74 ±17.15 | -16.97 ±23.08 | -4.69 ±20.84 | 12.29 ±19.52 | 0.7716 | 0.7395 | 0.2006 |
| **PYY (ng/mL x h)** | 0.67 ±0.25 | 0.86 ±0.32 | 0.46 ±0.34 | 1.09 ±0.40 | 0.76 ±0.33 | 0.6577 | 0.4995 | 0.0686 |

No significant interaction of body condition and diet were observed.

Values expressed as LSM±SEM

BC, body condition; GLP-1, glucagon-like peptide-1; GIP, gastric inhibitory polypeptide; LC, low carbohydrate; LF, low fat; LP, low protein; LSM, least square means; PYY, peptide YY; SEM, standard error of the mean
